# Investigating the Potential Hearing Impairment and Ototoxicity in Children up to Six Years With Cystic Fibrosis After Aminoglycoside Exposure (PIANO‐CF Extension)

**DOI:** 10.1002/ppul.27505

**Published:** 2025-02-11

**Authors:** Elena K. Schneider‐Futschik, Courtney B. Munro, Catherine Quinlan, Sarath Ranganathan

**Affiliations:** ^1^ Department of Biochemistry and Pharmacology, School of Biomedical Sciences, Faculty of Medicine, Dentistry and Health Sciences The University of Melbourne Parkville Victoria Australia; ^2^ Population Health, Murdoch Children's Research Institute Parkville Victoria Australia; ^3^ Kidney Regeneration Murdoch Children's Research Institute Melbourne Australia; ^4^ Department of Paediatrics, School of Medicine University of Melbourne Australia; ^5^ Department of Paediatric Nephrology Royal Children's Hospital Melbourne Australia; ^6^ Respiratory Medicine, Royal children's Hospital Melbourne Victoria Australia

## Abstract

**Background:**

People with CF (pwCF) are often treated with prolonged courses of aminoglycosides (AGs), for which known adverse effects include ototoxicity as a subset of hearing impairment (HI).

**Methods:**

The PIANO‐CF trial was a single‐center study conducted at The Royal Children's Hospital where 28 pediatric patients aged < 7 years underwent sequential hearing tests at increased range up to 12,500 Hz in relation to receiving intravenous (IV) AGs. More than 85% of the cohort (*n* = 24) participated in the follow‐up hearing testing up to 1 year.

**Results:**

HI was defined by degree (dB) and frequency (Hz) on the audiogram. This was further reviewed to determine if the type of HI was consistent with ototoxicity as there are frequently other causes of HI in this age group. At baseline the prevalence of HI and ototoxicity were 11% and 7%, respectively. Over a period of 1 year, HI was identified in 12.5% and that of ototoxicity in 6%. No correlation was found between degree of IV AG exposure and HI or ototoxicity.

**Discussion:**

The finding of HI in young children with CF, including in those with minimal IV AG exposure, has implications for CF services to proactively screen for HI. Undetected HI may compromise learning outcomes and given the age of children studied, this is not insignificant during the acquisition and development of language skills.

**Conclusion:**

Routine audiometric testing for pwCF up to 12,500 Hz or beyond may increase sensitivity in detection of ototoxicity and should be considered for use in screening, monitoring, and future research.

## 1 Introduction

There are many causes of hearing impairment (HI), one of which is the use of ototoxic drugs [1]. Exposure to ototoxic medications (e.g., IV aminoglycosides [AG], gentamicin and tobramycin) can cause cochleotoxicity (which typically affects frequencies within the conventional hearing range but may also extend to frequencies higher than 8000 Hz) that is often delayed and usually permanent [2, 3] and vestibulotoxicity (affecting balance). Ototoxicity is damage to the hearing and/or balance organs that occurs after exposure to medications or chemicals that affect the inner ear [4]. People with cystic fibrosis (pwCF) are at risk for AG‐induced ototoxicity due to frequent treatment with high doses of AGs [5, 6, 7, 8]. Hearing is not routinely assessed in pwCF [9, 10, 11]; however, the UK CF Trust suggests an annual pure tone audiometry (PTA) assessment due to exposure to IV AG [12]. While hearing assessment has traditionally been limited to 8000 Hz, testing at extended high frequencies (EHFs) designated as above 8000 Hz is important as impairment is associated with poorer speech‐in‐noise recognition [13] and is recommended for an effective ototoxicity monitoring program [14].

The rates of ototoxicity vary depending on the type and dosage of the AG. A survey of 24 trials of gentamicin, tobramycin, and amikacin showed that their mean frequencies of cochleototoxicity were 7.7%, 9.7% and 13.8%, respectively [15]. The frequency of AG‐induced cochleototoxicity with average doses of gentamicin, tobramycin, and amikacin of 3.9, 3.8, and 15.4 mg/kg, was 8.3%, 6.1%, and 13.9% for each, respectively [16]. These doses are significantly lower than average doses routinely used in children with CF [17]. At the Royal Children's Hospital, Melbourne, gentamicin, tobramycin and amikacin doses are significantly higher for children with CF at 7.5, 12, and 22.5 mg/kg, respectively.

The factors that determine an individual's susceptibility to AG‐induced ototoxicity include: individual tolerance, genetic predisposition (e.g., *MT‐RNR1* mutation), familial susceptibility, age extremes, daily dosage, duration and route of drug administration and possibly dosing strategy, long term exposure (i.e. cumulative dose) to ototoxic agents, impaired renal function and concomitant exposure to other ototoxic agents [2].

The literature to date shows the significance of AG toxicities including ototoxicity in people with CF. We considered the relationship between HI and IV AG exposure using lifetime IV AG exposure (measured in mg, mg/kg and number of days), lifetime IV gentamicin, tobramycin and amikacin exposure (in days) and lifetime IV AG courses (total number).

## 2 Methods

This study included 28 patients (ages 0−6 years, 16 males and 12 females) under active follow‐up at the Royal Children's Hospital from August 2014 and October 2018 (as part of the PIANO‐CF Extension study, HREC 33188E). The RCH Pediatric Audiology Department criteria were used to identify HI in more than 85% of the cohort (*n* = 24) participated in the follow‐up hearing testing up to 1 year (Supporting Information).

### 2.1 Study Type

This was a single‐center study in which ototoxicity in children with CF receiving an IV AG were investigated. Patient characteristics are found in Table 1. When receiving an IV AG, nebulized or inhaled AGs are routinely withheld.

**Table 1:** Characteristics of study cohort at baseline who consented for hearing testing.

Characteristic	Gentamicin	Tobramycin	Mean difference	*p* value^#^
			95% CI	
*n*	15	13		
Male sex *n* (%)	8 (53%)	8 (62%)		0.6674
CFTR genotype				
p.Phe508del homozygous *n* (%)	9 (60%)	6 (46%)		
p.Phe508del heterozygous *n* (%)	5 (33%)	6 (46%)		
Other *n*, (%)	1 (7%)	1 (8%)		
Pancreatic insufficient *n* (%)	15 (100%)	13 (100%)		
Age (years)	4.26 ± 1.76	3.69 ± 2.29	0.56 –1.05 to 2.17	0.4775
Weight (kg)	18.02 ± 4.97	16.39 ± 6.34	1.63 –2.86 to 6.12	0.4603
Weight centile	60.79 ± 21.73	55.18 ± 33.32	5.62 –16.84 to 28.07	0.6088
Weight Z‐score	0.31 ± 0.63	0.28 ± 1.36	–0.03 –0.83 to 0.90	0.9365
Height (cm)	103.55 ± 13.70	97.1 ± 21.12	6.43 –7.79 to 20.65	0.3579
Height centile	51.26± 22.20	51.37 ± 36.08	–0.11 –24.12 to 23.91	0.9926
Height *Z*‐score	–0.02 ± 0.63	0.05 ± 1.52	–0.03 –0.98 to 0.93	0.9533
BMI (m^2^)	16.30 ± 1.45	16.61 ± 1.89	–0.31 –1.65 to 1.02	0.6316
BMI centile	62.01 ± 25.29	57.25 ± 32.27	4.76 –18.09to 27.61	0.6712
BMI *Z*‐score	0.42 ± 0.0.82	0.34 ± 1.19	0.08 –0.73 to 0.89	0.8389
Also enrolled in COMBAT‐CF trial *n*, (%)	2 (13%)	2 (15%)		

*Note:* Mean ± SD. For normally distributeddata, an unpaired (two‐sample) *t*‐test with unequal variance and Welch approximation was used. For non‐parametric data, Wilcoxon rank sum (Mann−Whitney) was used.

### 2.2 Data Collection

Data were obtained from the patient's medical records. Thus, the analyses were based on clinical and laboratory data collected as part of the patients' regular medical care and inclusion criteria of the PIANO‐CF study (HREC 33188E). Of note, hearing tests were not routinely performed and so were performed as part of the study protocol in addition to standard care. Demographics (age ≤ 6 years and gender), serum creatinine levels, CF genotype, chronic infection status, body mass index (BMI), lung function, CFRD status, transplantation status and data on lifetime exposure to intravenous AG and oral macrolide use were recorded. Exclusion criteria were IV AG in the previous 8 weeks or IV immunoglobulin in the previous 12 weeks, respectively, pre‐existing renal impairment or (reduced eGFR calculated by the Schwartz equation) or a pre‐existing kidney condition (including, but not limited to, nephrotic syndrome, vesicoureteric reflux, urolithiasis and pyelonephritis) and diabetes mellitus [18].

### 2.3 Hearing Test

Participants had a baseline hearing test performed on admission before being given an IV AG. In instances where this was not possible (e.g., admission over a weekend), a baseline audiometric assessment was performed at the next available appointment, or the results of a previous hearing test were obtained. The hearing test was repeated at the child's next outpatient visit, usually four to 6 weeks post‐discharge and approximately 1 year later if the family had consented (this was an optional part of the study). The results of the hearing test were documented on RCH Audiological Investigations form MR481/I and stored in the medical records. A description of the types of audiometric assessment, or hearing tests, is provided below. Each test is painless, and the type of assessment performed was determined by the age and cooperation of the child. Audiometry was conducted blind to knowledge of AG treatment.

### 2.4 Definition of HI and Ototoxicity

As per definition by the World Health Organization (WHO), HI is defined if a person/adult is not able to hear as well as someone with normal hearing, meaning hearing thresholds of 20 dB or better in both ears. It can be mild, moderate, moderately severe, severe or profound, and can affect one or both ears [2, 19, 20]. The prevalence was defined as the number of cases of HI at baseline. Incidence was defined as the number of new cases over the study period. Since the WHO grading system for HI applies to adults, we used the widely accepted classification system from the Royal Children's Hospital Pediatric Audiology Department, which specializes in pediatric patients. HI was defined by occurrence of impairment > 20dB [21]. It was then defined by frequency (e.g., 8000 Hz v. 12,500 Hz) and location (e.g., right ear, left ear, or bilateral).

When HI was detected, the audiogram was reviewed by a senior pediatric audiologist to determine if changes were consistent with ototoxicity. Changes were considered consistent with ototoxicity if HI was at high frequencies (> 8000 Hz), testing reliability was good and tympanometry was classed as type A (healthy) in both ears. If impairment was at high frequencies, testing reliability was good and tympanometry was classed as type B or C (indicating possible congestion), it was uncertain whether changes in hearing were due to the presence of inner ear fluid, and this was considered “indeterminate.”

We considered the relationship between HI and IV AG exposure using lifetime IV AG exposure (in absolute mg, mg/kg and number of days), lifetime IV gentamicin, tobramycin, and amikacin exposure (in days) and lifetime IV AG courses (total number).

## 3 Results

We assessed the prevalence of HI at baseline before IV AGs exposure in children with CF. Twenty‐eight children consented for the hearing sub‐arm of the PIANO‐CF study, for whom 76 hearing tests were completed with a median of three tests per child. More than 85% of the cohort (*n* = 24) participated in follow‐up hearing testing up to 1 year. Details of the children whose hearing was tested are tabulated for the entire cohort and for each AG in Table 2. HI was observed in nine of the 76 tests (12%). There were five tests where HI was considered ototoxicity, and four tests were considered indeterminate. In these four tests, tympanometry results indicated eustachian tube dysfunction, which may have contributed to HI; thus, it is uncertain whether impairment was indeed ototoxicity. Of the study cohort, five children (18%) exhibited HI at any point in time. Some children whose hearing was impaired went on to subsequently have normal tests. The details of all children who displayed HI are shown in Table 3. All nine instances identified as HI were tested to a frequency of 12,500 Hz. The majority of cases were classified as mild impairment (*n* = 4) with only one child displaying a moderate degree of HI (Table 3).

**Table 2:** Number of hearing tests by time point and aminoglycoside.

Hearing Tests by time point	All AGs	Gentamicin	Tobramycin
At baseline	28	15	13
At next outpatient visit	24	12	12
At ≥ 1 year following study admission	24	12	12
Total	76	39	37

*Note:* The type of testing was determined by the child's age and most tests were play (*n* = 30) or PTA (*n* = 30), followed by VROA (*n* = 14), BOA (*n* = 1) and ASSR (*n* = 1).

**Table 3:** The results for the five children who demonstrated hearing impairment at any time point (event).

Study ID	Lifetime IV AG exposure^a^ (mg)	Lifetime courses of IV AG	Event	Hearing impairment	Degree of impairment	Frequency tested to	Location	Ototoxicity	Tympanometry
A	3570	3	Baseline	Yes	Mild	12,500 Hz	R	Yes	R: type A, L: type A
			Next Outpatient Visit	Yes	Mild	12,500 Hz	R	Yes	R: type A, L: type A
			One Year Follow‐up	No		8000 Hz			R: type A, L: type A
B	3720	2	Baseline	No		12,500 Hz			R: type A, L: type A
			Next Outpatient Visit	Yes	Mild	12,500 Hz	L	Yes	R: type A, L: type A
			One Year Follow‐up	No		12,500 Hz			R: type A, L: type A
C	8085	6	Baseline	Yes	Moderate	12,500 Hz	L	Indeterminate	R: type A, L: type B
			Next Outpatient Visit	Yes	Moderate	12,500 Hz	bilateral	Indeterminate	R: type C, L: type C
			One Year Follow‐up	Yes	Moderate	12,500 Hz	L	Indeterminate	R: type A, L: type C
D	495	2	Baseline	No		12,500 Hz			R: type A, L: type A
			Next Outpatient Visit	Yes	Mild	12,500 Hz	L	Yes	R: type A, L: type A
			One Year Follow‐up	No		8000 Hz			R: type A, L: type A
E	2688	1	Baseline	Yes	Mild	12,500 Hz	bilateral	Yes	R: type A, L: type A
			Next Outpatient Visit	No		12,500 Hz			R: type A, L: type A
			One Year Follow‐up	Yes	Mild	12,500 Hz	R	Indeterminate	R: type C, L: type C

^a^ Before first study‐related hearing test.

HI at baseline was observed on three occasions; two occasions were classified as ototoxicity, and one was indeterminate. This represents a prevalence of HI and ototoxicity at 11% and 7%, respectively. There were no differences between children who received gentamicin at baseline and those who received tobramycin (Table 1). Next, we studied the incidence of HI over the study period. Excluding baseline hearing tests, HI was observed on four occasions at the next outpatient visit and two occasions at the 1‐year follow‐up. This represents incidences of HI and ototoxicity of 12.5% and 6%, respectively, over a period of 1 year. There was no difference in HI or ototoxicity between those who received gentamicin and those given tobramycin (*χ* ^2^ *p* = 0.326 and *p* = 0.688, respectively).

Furthermore, we assessed if there was an association between HI and cumulative IV AG exposure. Regarding cumulative IV AG exposure two‐thirds had previous exposure (64%) and one‐third was AG naive (36%). In the children with previous exposure, the median number of IV AG courses was three, IQR one to six courses (range 1−11 courses). If IV AG exposure was examined using a categorical variable, the majority of children (61%) had one to four courses, followed by five to nine courses (33%) and only a one child (6%) had ≥ 10 courses. Data for IV AG exposure were skewed; therefore, non‐parametric analysis was performed using Spearman's rank correlation for all exposure variables. There was no association between HI and IV AG exposure for all variables representing IV AG exposure, as shown in Table 4.

**Table 4:** IV AG exposure non‐parametric data Spearman's rank correlation was used.

Characteristic	Mean	SD	Min/max
	28		
Lifetime AG exposure (mg)	3300	3816	0 to 11,317
Lifetime AG exposure (mg/kg)	256	279	0 to 848
Lifetime AG exposure (days)	29	33	0 to 108
Lifetime gentamicin exposure (days)	22	31	0 to 108
Lifetime tobramycin exposure (days)	7	11	0 to 41
Lifetime amikacin exposure (days)	0.4	2	0 to 11
Lifetime IV AG courses (#)	4	4.6	0 to 15

The same result was observed when specifically examining ototoxicity and cumulative IV AG exposure. The values of rho observed were all within the range of 0−0.2; this indicating very low correlation. These results indicate no linear relationship between HI or ototoxicity and cumulative IV AG exposure. Several children were exposed to oral macrolide antibiotics (azithromycin, clarithromycin, and erythromycin) in the 12 months preceding admission and recruitment into the study. None of these children had HI on any occasion. None of the patients receiving azithromycin had HI. The hearing of children with CF was not compared to that of healthy controls.

## 4 Discussion

In this study, we assessed the incidence of HI and ototoxicity in 28 pediatric patients with CF after acute exposure to IV AGs. The RCH Pediatric Audiology Department criteria were used to identify HI. The classification of pediatric HI varies across publications. The American Academy of Pediatics and the American Speech‐Language‐Hearing Association adopt comparable classifications. The RCH criteria are more rigorous than limits applied in most of the published literature and therefore, may underestimate the incidence and prevalence of HI and subsequently, ototoxicity.

Unique to this study was the investigation of the characteristics of HI observed and tympanometry results. This was done to ascertain whether the impairment observed was suggestive of ototoxicity, as opposed to conductive HI caused by fluid accumulation (e.g., as in a middle ear infection which is common in children). Previous studies have not separated HI and ototoxicity and thus, may incorrectly assume that all HI is ototoxicity.

The prevalence of HI at baseline was lower at 11% than that recently reported in a study in children with CF at 31.8%, although the cohort was comparatively younger and the classification of HI that we employed was more conservative [22]. It was, however, higher than the rate reported by CF data registries in children with CF of 0.9−1.1% [23, 24]. It is possible with increasing age that the likelihood of increased IV AG exposure, rates of HI are higher. Additionally, doses of AGs used in CF are higher than the general population and consequently rates of HI reported in product literature likely underreport HI in CF.

The incidence of HI reported represents the number of new cases of HI observed over the study period of approximately 1 year. During this time, three tests were performed: at baseline, at approximately four to 6 weeks after study admission for IV AG and at approximately 1‐year post‐admission. The only other study to examine hearing in children with CF longitudinally was TOPIC ‐once versus three‐times daily regimens of tobramycin treatment for pulmonary exacerbations of cystic fibrosis‐ trial [25]. The time profile of AG‐induced ototoxicity and the optimal time for detection of HI, specifically ototoxicity, is unknown. It is possible that testing at these time points may have missed acute transient changes in hearing or, conversely, long‐term cumulative toxicity and impairment that arises beyond 1 year.

Based on previous studies we hypothesized there would be a positive association between exposure (cumulative IV AG's, with a potential additive effect of oral azithromycin use) and ototoxicity. Hence, the lack of correlation between HI and cumulative IV AG exposure was surprising. One reason may be that our study population was younger than most previously studied and therefore had less IV AG exposure. Indeed, only one child had exposure to ≥ 10 courses of IV AGs. The study cohort was however, not without IV AG exposure with two‐thirds having previous exposure.

The finding of HI in young children with CF, including in those with minimal IV AG exposure, has implications for CF services to proactively screen for auditory impairment. Undetected, HI may compromise learning outcomes and given the age of children studied, this is not insignificant during the acquisition and development of language skills.

Research into AG‐induced ototoxicity has shifted in the last decade to focus on screening for mutations that may predispose patients to ototoxicity. Current work also concerns the use of concomitant otoprotectants to reduce the risk and rate of otoxicity, including aspirin, N‐acetylcysteine and D‐methionine [26, 27, 28, 29, 30] and developing less ototoxic AG antibiotics. This may change the incidence and prevalence of AG‐induced ototoxicity in the future.

### 4.1 Limitations

These finding may be somewhat limited by the low number of participants. Although interestingly these children still had significant IV AG exposure despite their young age. There is the possibility that there may be an association between cumulative drugs doses and ototoxicity, but that it is not a linear relationship and therefore could not be detected with the statistical testing applied in this study. Furthermore, we only collected data on IV AG exposure before the first hearing test, and not subsequently.

It is important to bear in mind there is potential bias as the type of testing performed was not uniform. This was due to the variability in age of our cohort (0−6 years) and the fact that audiological testing is guided by age and the child's developmental stage. Secondly, the limit of testing (frequency) must be considered in the interpretation of the outcome of HI. Most of the testing was performed up to 8000 Hz (*n* = 40, 53%), followed by testing performed to 12,500 Hz (*n* = 34, 45%), with extended high frequency testing above 8000 Hz preferred for the detection of ototoxicity. Testing was not always performed to 12,500 Hz on account of the equipment that was available at the time of the audiology appointment, therefore HI may have been overlooked in these patients. On two occasions (3%) for an infant, testing was performed to a frequency of 4000 Hz as owing to the child's age, PTA testing to 8000 or 12,500 Hz was not possible. Categories were used to classify the severity of HI as opposed to calculating changes in individual thresholds.

Furthermore, we did not test for the presence of a variation in *MT‐RNR1* which has been reported to make the individual more susceptible to AG‐induced ototoxicity [31, 32, 33].

There is debate as to what is considered the threshold of EHFs. We considered above 8000 Hz EHF and used the local RCH criteria for defining HI, as we were reliant on the clinical service performing audiometry. On nine occasions, HI was not detected at 8000 Hz, yet when tested at 12,500 Hz, there was HI (Table 5).

**Table 5:** Detection of hearing impairment if tested to 8000 Hz compared to 1250.

		500 Hz		1000 Hz		2000 Hz		4000 Hz		8000 Hz			12,500 Hz		
Study ID	Event	R	L	R	L	R	L	R	L	R	L	HI at 8000 Hz	R	L	HI at 12,500 Hz
A	Baseline	15	15	10	10	10	10	5	5	5	5	No	30	20	Yes
	Next outpatient	20	15	10	10	5	5	5	0	10	15	No	20	30	Yes
B	Next outpatient	10	10	5	5	10	5	0	0	10	15	No	20	25	Yes
C	Baseline	20	15	10	10	10	10	15	15	10	15	No	20	55	Yes
	Next outpatient	10	15	10	20	0	5	0	5	0	10	No	35	60	Yes
	One year	5	15	5	25	0	0	0	0	0	0	No	20	50	Yes
D	Next outpatient	10	5	5	5	0	0	5	0	0	0	No	10	25	Yes
E	Baseline	5	10	0	5	0	5	5	5	5	5	No	30	30	Yes
	One year	15	15	10	5	0	0	5	10	5	5	No	25	20	Yes

### 4.2 Recommendations

Concerning the findings of this study and the current evidence base, baseline audiometric assessment of all persons with CF is recommended before first IV AG exposure [34]. Testing using EHFs up to, or beyond, 12,500 Hz is advocated, as 8000 Hz may fail to detect AG‐induced ototoxicity. Future studies should prospectively measure hearing at baseline and changes with ongoing IV AG exposure. There is no well‐established threshold of IV aminoglycoside exposure in pediatric or adult patients that reliably predicts the risk of ototoxicity. In fact, in adult CF patients, one course of IV tobramycin was sufficient to cause HI and other ototoxic symptoms 4 weeks after treatment ended [35, 36]. Dong et al. showed that their advanced pharmacokinetic modeling is able to provide an informed analysis predicting ototoxicity risk in patients with CF treated with tobramycin [6]. Hence, as most adult patients with CF are frequently exposed to intravenous AG, resulting in HI at a rate significantly higher than what would be expected from aging alone [37].

### 4.3 Clinical Applications

Early detection may lead the clinician to consider alternative treatment to prevent ongoing HI. For example, choosing nebulized antibiotics rather than IV, which have a greater surface area deposition in lungs, lower systemic absorption, and lower incidence of adverse effects like ototoxicity. The therapeutic potential for ameliorating HI with concomitant use of otoprotectants such as N‐acetyl cysteine, D‐methionine or glutathione may offer future benefits, but both the patient population who would benefit most and the clinical efficacy of these regimens are currently ill‐defined and more work needs to be done to ensure lack of interference between the otoprotectant with the AGs' antibacterial activity.

### 4.4 Conclusion

The prevalence of HI and ototoxicity were 11% and 7%, respectively. Over a period of 1 year, the incidence of HI was 12.5% and that of ototoxicity was 6%. No correlation was found between IV AG exposure and HI or ototoxicity. Testing to 12,500 Hz increased sensitivity for detection of ototoxicity and as such testing in the higher ranges should be considered for use in screening, monitoring and future research.

In defining a protocol for assessment of ototoxicity in children with CF, the purpose of the assessment should be considered (e.g., screening or monitoring) with regard to the target population (e.g., age group, exposure status, current treatments). There should be a system for referral with selection of appropriate audiometric tests for AG‐induced ototoxicity and developmental age and stage. This should consider the schedule or frequency of testing in relation to IV AG exposure and the definition of a significant change in hearing. A clear communication pathway for sharing audiometric test results and discussing the frequency of follow‐up in relation to the patient's antibiotic regimen should be established. Multi‐disciplinary approaches such as advocated by the CF Foundation provide recommendations to navigate decisions related to otolaryngology consultation [38].

## Author Contributions

**Elena K. Schneider‐Futschik:** writing–original draft, writing–review and editing, validation, software, investigation. **Courtney B. Munro:** conceptualiztion, funding acquisition, project administration, writing–review and editing, visualisation. **Catherine Quinlan:** methodology, data curation, supervision, resources. **Sarath Ranganathan:** conceptualization, methodology, investigation, validation, formal analysis, supervision, funding acquisition, visualization, project administration, resources, writing–review and editing.

## Ethics Statement

Ethics approval was obtained by the Royal Children's Hospital (HREC 33188E). We recognize that standards have been followed (Declaration of Helsinki; US Federal Policy for the Protection of Human Subjects; or European Medicines Agency Guidelines for Good Clinical Practice). All persons involved have provided their informed consent before inclusion in the study.

## Conflicts of Interest

The authors declare no conflicts of interest.

## Supporting information

Supporting information.

## Data Availability

The data that support the findings of this study are available from the corresponding author upon reasonable request. Some data may not be made available because of privacy or ethical restrictions.

## References

[1] G. Paludetti , G. Conti , W. DI Nardo , et al., “Infant Hearing Loss: From Diagnosis to Therapy Official Report of XXI Conference of Italian Society of Pediatric Otorhinolaryngology,” Acta Otorhinolaryngologica Italica: Organo Ufficiale Della Societa Italiana Di Otorinolaringologia E Chirurgia Cervico‐facciale 32, no. 6 (December 2012): 347–370. 23349554 PMC3552543

[2] Organization. WH . Programme for the Prevention of Deafness and Hearing Impairment,” In Report of An Informal Consultation on Strategies for Prevention of Hearing Impairments from Ototoxic Drugs. (World Health Organization, 1995), 21–23.

[3] M. Jiang , T. Karasawa , and P. S. Steyger , “Aminoglycoside‐Induced Cochleotoxicity: A Review,” Frontiers in Cellular Neuroscience 11 (2017): 308, 10.3389/fncel.2017.00308. 29062271 PMC5640705

[4] Accessed December, 2024, https://www.audiology.org/consumers-and-patients/hearing-and-balance/ototoxicity/.

[5] C. R. Schubert , A. J. Paulsen , D. M. Nondahl , et al., “Association Between Cystatin C and 20‐Year Cumulative Incidence of Hearing Impairment in the Epidemiology of Hearing Loss Study,” JAMA Otolaryngology–Head & Neck Surgery 144, no. 6 (June 2018): 469–474, 10.1001/jamaoto.2018.0041. 29710267 PMC6014887

[6] M. Dong , A. V. Rodriguez , C. A. Blankenship , G. McPhail , A. A. Vinks , and L. L. Hunter , “Pharmacokinetic Modelling to Predict Risk of Ototoxicity With Intravenous Tobramycin Treatment in Cystic Fibrosis,” Journal of Antimicrobial Chemotherapy 76, no. 11 (October 2021): 2923–2931, 10.1093/jac/dkab288. 34379758 PMC8677449

[7] A. C. Garinis , C. P. Cross , P. Srikanth , et al., “The Cumulative Effects of Intravenous Antibiotic Treatments on Hearing in Patients With Cystic Fibrosis,” Journal of Cystic Fibrosis 16, no. 3 (May 2017): 401–409, 10.1016/j.jcf.2017.01.006. 28238634 PMC5591745

[8] C. M. Blankenship , L. L. Hunter , M. P. Feeney , et al., “Functional Impacts of Aminoglycoside Treatment on Speech Perception and Extended High‐Frequency Hearing Loss in a Pediatric Cystic Fibrosis Cohort,” American Journal of Audiology 30, no. 3S (October 2021): 834–853, 10.1044/2020_AJA-20-00059. 33465313 PMC9126133

[9] R. Abusamra and D. McShane , “Is Deafness Mutation Screening Required in Cystic Fibrosis Patients?,” Paediatric Respiratory Reviews 20, no. Suppl (August 2016): 24–26, 10.1016/j.prrv.2016.06.010. 27427311

[10] R. Abusamra and A. Gupta , “National Survey of Hearing Assessment in Patients With Cystic Fibrosis in the UK,” European Respiratory Journal 48, no. suppl 60 (2016): PA1260, 10.1183/13993003.congress-2016.PA1260.

[11] Z. Farzal , Y. F. Kou , R. St. John , G. B. Shah , and R. B. Mitchell , “The Role of Routine Hearing Screening in Children With Cystic Fibrosis on Aminoglycosides: A Systematic Review,” The Laryngoscope 126, no. 1 (January 2016): 228–235, 10.1002/lary.25409. 26152803

[12] Group. UCFTAW . Antibiotic Treatment For Cystic Fibrosis. 2009, www.cysticfibrosis.org.uk/the-work-we-do/clinical-care/consensus-documents.

[13] S. K. Mishra , U. Saxena , and H. Rodrigo , “Hearing Impairment in the Extended High Frequencies in Children Despite Clinically Normal Hearing,” Ear & Hearing 43, no. 6 (November/December 2022): 1653–1660, 10.1097/AUD.0000000000001225. 35470812

[14] A. C. Garinis , D. H. Keefe , L. L. Hunter , et al., “Chirp‐Evoked Otoacoustic Emissions and Middle Ear Absorbance for Monitoring Ototoxicity in Cystic Fibrosis Patients,” Ear & Hearing 39, no. 1 (January/February 2018): 69–84, 10.1097/AUD.0000000000000464. 28708814 PMC5741529

[15] J. K. Aronson , ed., Aminoglycoside.” In Antibiotics. Meyler's Side Effects of Drugs: The International Encyclopedia of Adverse Drug Reactions and Interactions (The Netherlands: Elsevier, 2006. 1, 15th ed., 118–136).

[16] G. Kahlmeter and J. I. Dahlager , “Aminoglycoside Toxicity—A Review of Clinical Studies Published between 1975 and 1982,” Journal of Antimicrobial Chemotherapy 13, no. Suppl A (January 1984): 9–22, 10.1093/jac/13.suppl_a.9. 6365884

[17] J. Massie and N. Cranswick , “Pharmacokinetic Profile of once Daily Intravenous Tobramycin in Children With Cystic Fibrosis,” Journal of Paediatrics and Child Health 42, no. 10 (October 2006): 601–605, 10.1111/j.1440-1754.2006.00944.x. 16972966

[18] J. B. Levy , “Nephrotoxicity of Intravenous Immunoglobulin,” QJM: Monthly Journal of the Association of Physicians 93, no. 11 (November 2000): 751–755, 10.1093/qjmed/93.11.751. 11077032

[19] https://www.who.int/health-topics/hearing-loss#tab=tab_1.

[20] https://www.who.int/publications/i/item/9789240020481.

[21] K. Graydon , G. Rance , R. Dowell , and B. Van Dun , “Consequences of Early Conductive Hearing Loss on Long‐Term Binaural Processing,” Ear & Hearing 38, no. 5 (September/October 2017): 621–627, 10.1097/AUD.0000000000000431. 28353521

[22] K. L. Kreicher , M. J. Bauschard , C. S. Clemmens , C. M. Riva , and T. A. Meyer , “Audiometric Assessment of Pediatric Patients With Cystic Fibrosis,” Journal of Cystic Fibrosis 17, no. 3 (May 2018): 383–390, 10.1016/j.jcf.2017.10.007. 29289454

[23] 2016 CFTUCFRADR. (2017): 1–60, www.cysticfibrosis.org.uk/the-work-we-do/uk-cf-registry/reporting-and-resources.

[24] N. Soulsby , S. Bell , H. Greville , and C. Doecke , “Intravenous Aminoglycoside Usage and Monitoring of Patients With Cystic Fibrosis in Australia. What's New?,” Internal Medicine Journal 39, no. 8 (August 2009): 527–531, 10.1111/j.1445-5994.2008.01787.x. 19220547

[25] A. Smyth , K. H. V. Tan , P. Hyman‐Taylor , et al., “Once Versus Three‐Times Daily Regimens of Tobramycin Treatment for Pulmonary Exacerbations of Cystic Fibrosis—The Topic Study: A Randomised Controlled Trial,” The Lancet 365, no. 9459 (February 2005): 573–578, 10.1016/S0140-6736(05)17906-9. 15708100

[26] D. J. Fox , M. D. Cooper , C. A. Speil , et al., “D‐Methionine Reduces Tobramycin‐Induced Ototoxicity Without Antimicrobial Interference in Animal Models,” Journal of Cystic Fibrosis 15, no. 4 (July 2016): 518–530, 10.1016/j.jcf.2015.06.005. 26166286 PMC4706822

[27] L. Feldman , R. A. Sherman , and J. Weissgarten , “N‐Acetylcysteine Use for Amelioration of Aminoglycoside‐Induced Ototoxicity in Dialysis Patients,” Seminars in Dialysis 25, no. 5 (September/October 2012): 491–494, 10.1111/j.1525-139X.2012.01090.x. 22708712

[28] M. E. Huth , A. J. Ricci , and A. G. Cheng , “Mechanisms of Aminoglycoside Ototoxicity and Targets of Hair Cell Protection,” International Journal of Otolaryngology 2011 (2011): 937861, 10.1155/2011/937861. 22121370 PMC3202092

[29] A. Prayle , A. Watson , H. Fortnum , and A. Smyth , “Side Effects of Aminoglycosides on the Kidney, Ear and Balance in Cystic Fibrosis,” Thorax 65, no. 7 (July 2010): 654–658, 10.1136/thx.2009.131532. 20627927 PMC2921289

[30] K. Kranzer , W. F. Elamin , H. Cox , J. A. Seddon , N. Ford , and F. Drobniewski , “A Systematic Review and Meta‐Analysis of the Efficacy and Safety of N‐Acetylcysteine in Preventing Aminoglycoside‐Induced Ototoxicity: Implications for the Treatment of Multidrug‐Resistant TB,” Thorax 70, no. 11 (November 2015): 1070–1077, 10.1136/thoraxjnl-2015-207245. 26347391

[31] G. Al‐Malky , R. Suri , T. Sirimanna , and S. J. Dawson , “Normal Hearing in a Child With the m.1555A>G Mutation Despite Repeated Exposure to Aminoglycosides. Has the Penetrance of This Pharmacogenetic Interaction Been Overestimated?,” International Journal of Pediatric Otorhinolaryngology 78, no. 6 (June 2014): 969–973, 10.1016/j.ijporl.2014.02.015. 24703164

[32] S. Rahman , R. Ecob , H. Costello , et al., “Hearing in 44‐45 Year Olds With m.1555A>G, a Genetic Mutation Predisposing to Aminoglycoside‐Induced Deafness: a Population Based Cohort Study,” BMJ Open 2, no. 1 (2012): e000411, 10.1136/bmjopen-2011-000411. PMC3253422 22223843

[33] G. Al‐Malky , S. J. Dawson , T. Sirimanna , E. Bagkeris , and R. Suri , “High‐Frequency Audiometry Reveals High Prevalence of Aminoglycoside Ototoxicity in Children With Cystic Fibrosis,” Journal of Cystic Fibrosis 14, no. 2 (March 2015): 248–254, 10.1016/j.jcf.2014.07.009. 25127922

[34] A. Garinis , D. Konrad‐Martin , and N. Bramhall , “Ototoxicity and Noise Damage: From Preclinical Findings to Audiological Management,” American Journal of Audiology 30, no. 3S (October 2021): 797–799, 10.1044/2021_AJA-21-00153. 34606329 PMC9126117

[35] E. E. Harruff , J. Kil , M. G. T. Ortiz , et al., “Ototoxicity in Cystic Fibrosis Patients Receiving Intravenous Tobramycin for Acute Pulmonary Exacerbation,” Journal of Cystic Fibrosis 20, no. 2 (March 2021): 288–294, 10.1016/j.jcf.2020.11.020. 33341407

[36] M. A. Gleser and E. M. Zettner , “Negative Hearing Effects of a Single Course of IV Aminoglycoside Therapy in Cystic Fibrosis Patients,” International Journal of Audiology 57, no. 12 (December 2018): 923–930, 10.1080/14992027.2018.1514537. 30382794

[37] E. M. Zettner and M. A. Gleser , “Progressive Hearing Loss Among Patients With Cystic Fibrosis and Parenteral Aminoglycoside Treatment,” Otolaryngology–Head and Neck Surgery 159, no. 5 (November 2018): 887–894, 10.1177/0194599818782444. 29914288

[38] A. J. Kimple , B. A. Senior , E. T. Naureckas , et al., “Cystic Fibrosis Foundation Otolaryngology Care Multidisciplinary Consensus Recommendations,” International Forum of Allergy & Rhinology 12, no. 9 (September 2022): 1089–1103, 10.1002/alr.22974. 35089650 PMC9545592

