# Supplementary material for: Investigating the Potential Hearing Impairment and Ototoxicity in Children up to Six Years With Cystic Fibrosis After Aminoglycoside Exposure (PIANO‐CF Extension)
Source: Pediatr Pulmonol. 2025 Feb 11;60(2):e27505. doi: 10.1002/ppul.27505 (PMC11814909; doi:10.1002/ppul.27505)
Supplement: Supplementary file 1 — Supporting information. [file PPUL-60-0-s001.docx]

**SUPPLEMENTARY INFORMATION**

**Investigating the potential Hearing Impairment and Ototoxicity in children up to six years with Cystic Fibrosis after Aminoglycoside Exposure (PIANO-CF extension)**

**Running Title:** Ototoxicity after Aminoglycoside Exposure in Cystic Fibrosis

Elena K. Schneider-Futschik^1*^, Courtney B. Munro^2,3,4*^, Catherine Quinlan^2,3,4^, Sarath Ranganathan^#^

**Supplementary Information**

| **Description of hearing levels** | **RCH** | **Healthy Hearing Program^1^** | **Deaf Children Australia^2^** | **^3^** |
| --- | --- | --- | --- | --- |
|  | **Threshold range in dBHL** | | | |
| Normal | 0-20 | 10-20 | 0-20 | 0-20 |
| Mild hearing impairment | 25-40 | 21-40 | 20-41 | 20-39 |
| Moderate hearing impairment | 45-60 | 41-55 | 42-75 | 40-69 |
| Moderate-severe |  | 56-70 |  |  |
| Severe hearing impairment | 65-85 | 71-90 | 76-90 | 70-89 |
| Profound hearing impairment | 90-110 | >90 | 91+ | >90 |

**References**

1. <https://www.childrens.health.qld.gov.au/__data/assets/pdf_file/0035/174968/hh-audiology-protocol.pdf>.

2. <https://www.deafchildrenaustralia.org.au/wp-content/uploads/2021/06/degrees-hearing-loss.pdf>.

3. <https://www.ncbi.nlm.nih.gov/books/NBK538285/#:~:text=Mild%20hearing%20loss%20corresponds%20to> teome.
